# Analyzing time-varying trip distributions with a random-effect spatial OD dependence model

**DOI:** 10.1371/journal.pone.0280162

**Published:** 2023-01-17

**Authors:** Linglin Ni, Xiaokun (Cara) Wang, Xiqun (Michael) Chen, Dapeng Zhang

**Affiliations:** 1 Beijing Wuzi University Logistics school, Beijing, China; 2 Department of Civil and Environmental Engineering, Rensselaer Polytechnic Institute, Troy, New York, United States of America; 3 College of Civil Engineering and Architecture, Zhejiang University, Hangzhou, China; 4 Alibaba-Zhejiang University Joint Research Institute of Frontier Technologies, Hangzhou, China; 5 School of Public Administration and Policy, Renmin University of China, Beijing, China; Texas A&M Transportation Institute, UNITED STATES

## Abstract

This paper proposes a random-effect spatial OD (origin-destination) dependence model to investigate varying trip distributions over time. By proposing a maximum likelihood estimation with spectral decomposition methods, the effects of spatial dependences and the unobservable zonal heterogeneity at the origin and destination can be estimated simultaneously. A series of numerical experiments and a real-world trip distribution study with cellular signaling data collected in Hangzhou, China, are conducted. This paper enriches the existing literature by developing (1) an innovative specification to allow for random effects in existing spatial OD dependence models; (2) an innovative estimation method to obtain the values of parameters and improve model fittings; and (3) a set of numerical experiments and an empirical trip distribution analysis that jointly captures spatial effects (spatial interaction and spatial OD dependences), and the unobservable zonal heterogeneity. This paper can equip policymakers with an effective tool for analyzing the OD travel flow over time which is a groundwork for making appropriate transportation policies.

## 1. Introduction

Many abnormal and disruptive events, like the COVID-19 (coronavirus disease 2019), can reshape travel patterns [1, 2] in urban [3, 4], suburban [5], and rural areas [6]. Understanding the day-to-day variation of travel patterns is one of the most important steps in responding to these circumstances. Unlike most traditional travel analyses that focus on long-term static patterns, analyses of travel patterns during abnormal events require a thorough understanding of time-varying effects. Properly attributing the fluctuation in trip distributions to known interventions or unknown effects is a trending and important research question.

Meanwhile, spatial analyses of travel patterns are necessary. During an abnormal event (e.g., COVID), some government agencies have imposed lockdown policies depending on the locations of outbreaks, restricting people from moving beyond a certain boundary. For example, certain Chinese cities applied community grid management policies during the outbreaks to regulate mobility. People can only go to designated places by a certain transportation mode in a less frequent manner. Spatial interaction models are needed to reveal when, where, and why people move across certain geographic regions. Overall, it is crucial to analyze residents’ travel patterns using spatial interaction models that recognize the time-varying effect. Such understanding is necessary for public agencies to enforce lockdown restrictions while providing essential life support.

However, the tool of analyzing time-varying trip distributions has not been sufficiently developed yet. Apart from known interventions, the trip number fluctuation at different points in time may come from two processes: one is the unobserved zonal heterogeneity driven by zonal characteristics and time invariant; The other is the unobserved idiosyncratic effects which can vary at different points in time across zones. Separating these two processes would improve the understanding of trip distribution fluctuation over time and lead to an efficient coefficient estimation from a random-effect model. Hence, given the societal importance, this paper develops a random-effect spatial OD dependence model to cater for the need of analyzing time-varying trip distributions. Two zonal error components (one for the origin zone and the other for the destination zone) in addition to the idiosyncratic error are specified to capture zonal heterogeneity of the origin and the destination. The developed model can jointly capture the unobservable zonal heterogeneity of the origin and the destination and establish their relationships with spatial OD dependences to improve the models’ fitting. Results can provide actuated elasticities of interventions, zonal heterogeneity, and strengths of spatial OD dependences.

Another notable innovation of this paper is in the manipulation of the variance-covariance matrix of trip flows for model estimation. Due to the existence of unobservable zonal heterogeneity, the construction of variance-covariance matrices becomes complicated. Directly maximizing the likelihood function would lead to a long computing time and sometimes be infeasible due to the limitation of computers’ memory. The essential elements of the computational hassle are the mathematical derivations of the inverse and determinants of the variance-covariance matrix. To tackle this computational issue, this paper borrows a spectral decomposition trick developed by Wansbeek and Kapteyn [7] to reduce the mathematic complexity [8]. This paper extends the spectral decomposition method by accounting for spatial OD dependences in the transformation of the variance-covariance matrix to arrive at a simplified log-likelihood function, enabling an efficient maximum likelihood estimation. The coefficients, spatial OD dependence parameters, and the variances of the error components can be obtained from maximizing the simplified likelihood function given the spectral decomposition.

A series of estimation experiments are followed to demonstrate the performance of the proposed estimation method. Using simulated data, this paper examines the scenario when the time-varying trip distributions can be attributed to both the explanatory variables and the unobservable random effects. Then, a real-world trip distribution analysis based on cellular signaling data is presented to examine the scenario when the difference of traffic volume at morning peaks are driven by the different components in unobservable random effects. The numerical experiments and the real-world application show the proposed model is an effective tool for analyzing the OD travel flow over time which is a groundwork for making appropriate transportation policies.

The next section will review the relevant literature to the proposed model. Then, model specifications, estimation methods, and numerical experiments are discussed in Section 3 and Section 4, respectively. Finally, the real-world study using the proposed model is conducted as an example to demonstrate the model’s applicability in Section 5 and is followed by conclusions drawn in Section 6.

## 2. Literature review

### 2.1 Spatial dependences in trip distribution

Spatial dependences have been recognized and analyzed in many studies. Properly capturing the effect of spatial dependences is vital for capturing the true relationship between attributes and travel patterns [9]. As Anselin and Griffith [10] proposed, ignoring spatial dependences would result in failure or inaccuracy of results, including inconsistent coefficients, misestimated standard errors, and under-performed model fittings. Bhat and Zhao [11] disclosed that it is important to study the household shopping stop-making behavior by accommodating spatial heterogeneity, spatial autocorrelation, and heteroscedasticity. Celik and Guldmann [12] also proposed that the consideration of adjacency should be included in models, in addition to distance in the explanation of travel patterns. Paleti et al. [13] explored the factors impacting vehicle ownership and travel mode choices by considering spatial dependences, using the 2009 National Household Travel Survey in California.

Furthermore, studies found that the spatial dependences of urban travel flow may be generated at both the origin and destination. For example, González et al. [14] explored that the travel mode choice of residents was determined by the zones’ characteristics between the origin and destination. LeSage and Pace [15] and Fischer and Griffith [16] disclosed that the spatial autocorrelation across commodity flows was the strongest when both origin and destination were located close to a highway network, and concluded that spatial effects were generated by the origin and destination. LeSage and Thomas-Agnan [17] further forecasted commodity flows among 15 Spanish regions by building a dynamic econometric model with the spatial OD dependences. Following the proposition of these models, a series of the existing literature has adopted the so-called spatial OD dependence specifications to analyze problems in transportation [18, 19] and commodity flow [20, 21]. These studies verified the necessity of capturing spatial OD dependences in spatial interaction models.

In summary, spatial OD dependence analyses are popular analytical tools to investigate travel flow. Various studies have demonstrated the necessity, applicability, and urgency of using these models. However, analyses combining spatial OD dependences and temporal effects are scarce, which is the research area this paper aims to enrich.

### 2.2 Random effects in trip distribution

Various technics can be deployed to address temporal effects in travel pattern studies. For example, Zhang and Wang [22] built a spatiotemporal regression model to disclose the dynamic spillover effects of low-cost airlines on airport airfare. In the context of panel trip distribution data, one of the important technics is random-effect analysis. Existing studies have widely adopted random effects in panel data models. For example, Dong et al. [23] built a spatial random slope standard multilevel modeling to explore travel satisfaction in Beijing, China. Huang et al. [24] built a random-effect ordered probit model to disclose that transit accessibility was negative with auto ownership based on the data of Guangzhou between 2011 and 2012. Hou et al. [25] built a negative binomial model with random effects to estimate the spatial relationships within groups.

However, in analyses of spatial OD dependences, random effects have not been considered. Notable research with a similar model specification is LeSage and Fischer [26], in which a fix-effect approach was used to process panel data. However, fixed effects cannot capture the unobservable zonal heterogeneity in the trip distribution data effectively. Specifically, for each origin or destination, a fixed-effect model specifies an intercept for each origin and destination zone. The fixed-effect terms are estimated similarly to explanatory variables. Such a treatment is powerful when the focus is on deriving a common effect size for identical origins and destinations and estimating time-varying explanatory variables. However, for analyzing day-to-day trip distribution variations, the explanatory variables are likely constants that cannot be identified when using fix-effect approaches. Meanwhile, the goal of the studies is often to generalize trip distribution patterns across regions and time.

### 2.3 Cellular signaling panel data in travel flow analyses

Traditional data collection for travel analyses is survey, which often runs into a lengthy process to collect movement information of residents, vehicles, and cargoes. Nonetheless, only a tiny proportion of all trips taking place in a city are surveyed. With the development of mobile communication technologies, researchers could use the massive cellular signaling data to analyze travel flow identification [27–32], Cellular signaling data have the advantages of high sampling rates, low collection costs, on-time updating frequencies, and accurate space-time information. For instance, Asakura and Hato [33] explored individual travel behavior in urban areas based on the travelers’ locations from their mobile phones. González et al. [14] found a high temporal and spatial regularity of human travel patterns using the six-month tracking data from 100,000 anonymized mobile phone users. Ahas et al. [34] explored the daily pattern and spatial differences of urban travel flows, using a 15-min intervals mobile telephone positioning data during 8 days of 277 respondents. Çolak et al. [28] used billions of mobile phone trajectories to analyze the pattern of people moving across cities. Calabrese et al. [35] used the mobile phone traces with millions of users living in a metropolitan area to disclose individual mobility patterns. Iqbal et al. [29] used a more than 1-month mobile phone call detail records from 2.87 million users of Dhaka, Bangladesh, to generated OD matrices.

In terms of the OD travel flow estimation, many researchers used cellular data to generate OD flows. For example, Aguiléra et al. [36] estimated travel times, train occupancy levels, and OD flows using cellular data in Paris. Zhang et al. [37] used cellular probe data to estimate the daily O-D demand with the Horvitz-Thompson estimator. Wang et al. [32] used the 6-week cellular data in the Kansas Metro Corridor to estimate the dynamic OD flow, travel demand data, and commuting flow. Ni et al. [19] used the cellular signal data in Hangzhou to investigate the effects of population, facilities, and transit accessibility on OD travel flows. These studies have shown that cellular signaling data, either disaggregated or aggregated, provided the basis for exploring and analyzing trip distribution. It’s compelling when the observed travel flows data is collected at different times, leading to panel data.

### 2.4 Summary of research gaps and objectives

In response to the current COVID-19 and future abnormal events, deepening the understanding of the trip distribution problem becomes urgent and necessary. In addition to traditional spatial analyses, capturing the unobservable zonal heterogeneity in trip distribution variation is a powerful tool for understanding time-varying effects. The gap of the existing literature is that there has not been a model to characterize spatial OD dependences and the unobservable zonal heterogeneity jointly. Given the availability of data collected at different times from cellular signals, developing such an innovative model becomes feasible and can empower the research community to examine the time-varying effects in trip distributions. Hence, this paper aims to specify, estimate, and apply an innovation model to understand time-varying trip distributions to make scientific and methodological contributions to the existing literature.

## 3. Methodology

Given the need of analyzing time-varying trip distributions in response to the unprecedented pandemic, a random-effect spatial OD dependence model is proposed to analyze the OD travel flow over time. The proposed econometric model can reveal the sensitivity of explanatory variables in interest to time-varying traffic volume, empowering transport planners to address traffic congestions. All data provided to this paper does not contain any individual level of information, which does not require ethical approval for the study.

### 3.1 Model specification

Let ***y***_***klt***_ be the number of trips between the origin, denoted as *k*(1…*K*), and destination, denoted as *l*(1…*L*), at time *t*(1…*T*). The model takes the form

$$y_{klt}=\rho_{o}I_{T}⊗\left(W_{o}⊗I_{L}\right)y_{klt}+\rho_{d}I_{T}⊗\left(I_{K}⊗W_{d}\right)y_{klt}+\rho_{w}\left(I_{T}⊗W_{w}\right)y_{klt}+X\beta +\alpha_{k}+\gamma_{l}+\varepsilon_{klt}$$ (1)

The number of trips ***y***_***klt***_ is of size (*KT* × *L*) and arranged as

$$y_{kl1}={\left[\begin{array}{ccc} y_{111} & y_{121} & \begin{array}{cc} \ldots & y_{1L1} \end{array}\\ y_{211} & y_{221} & \begin{array}{cc} \ldots & y_{2L1} \end{array}\\ \begin{array}{c} \vdots \\ y_{K11} \end{array} & \begin{array}{c} \vdots \\ y_{K21} \end{array} & \begin{array}{cc} \begin{array}{c} \ddots \\ \cdots \end{array} & \begin{array}{c} \vdots \\ y_{KL1} \end{array} \end{array} \end{array}\right]}_{\left(K\times L\right)}$$ (2)

and

$$y_{klt}={\left[\begin{array}{c} y_{kl1}\\ y_{kl2}\\ \begin{array}{c} \vdots \\ y_{klT} \end{array} \end{array}\right]}_{\left(KT\times L\right)}$$ (3)

The first spatial autoregressive term *ρ*_*o*_***I***_***T***_⨂(***W***_***o***_⨂***I***_***L***_)***y***_***klt***_ captures the spatial dependence of origins’ neighbors on the number of trips between origins and destinations, in which ***W***_***o***_ is a (*K* × *K*)-dimensional spatial weight matrix, ⨂ denotes the Kronecker product, and *I*_*s*_ (*s* = *K*,*L*,*T*) is the *s*-dimensional identity matrix. The coefficient *ρ*_*O*_ measures the strength of the spatial dependence at the origin. The second spatial autoregressive term *ρ*_*d*_***I***_***T***_⨂(***I***_***K***_⨂***W***_***d***_)***y***_***klt***_ allows for spatial dependence of destinations’ neighbors on the number of trips, and ***W***_***d***_ is a (*L* × *L*)-dimensional matrix. The coefficient *ρ*_*d*_ measures the strength of the dependence at the destination. The third spatial autoregressive term *ρ*_*w*_ (***I***_***T***_⨂***W***_***w***_)***y***_***klt***_ captures the dependence of OD travel flow which can be perceived as an interaction effect of the origins’ dependence and the destinations’ dependence. The matrix ***W***_***w***_ = ***W***_***o***_⨂***W***_***d***_ and *ρ*_*w*_ is the corresponding coefficient. There are various specifications of spatial weight matrices in empirical studies [38]. Throughout this paper, the elements in spatial weight matrices are the inverse distance between the centroids of origin and destination zones, and the matrices are row standardized.

Explanatory variables ***X*** contains the covariates at origin ***X***_***o***_ and destination ***X***_***d***_, such as populations, the number of jobs, and transportation accessibility metrics. ***X*** also involves OD interaction covariates ***X***_***od***_ such as distance between origin and destination. These are the common variables examined in spatial interaction models. Term ***β*** = [***β***_***o***_, ***β***_***d***_, ***β***_***od***_] are the corresponding coefficients to be estimated.

In the error term, ***α***_***k***_ denotes the unobservable origin’s effect and is destination- and time-invariant. ***γ***_***l***_ denotes the unobservable destination’s effect and is origin- and time-invariant. ***α***_***k***_ and ***γ***_***l***_ constitute a two-way error structure, which is a common specification for panel data modeling. Assume ***α***_***k***_ and ***γ***_***l***_ are random variables and independently and identically distributed across samples, i.e.

$$\alpha_{k}\sim N\left(0,\sigma_{\alpha }^{2}\right)$$ (4)

$$\gamma_{l}\sim N\left(0,\sigma_{\gamma }^{2}\right)$$ (5)

leading to a random-effect model. The variances can be obtained in the maximum likelihood estimation along with the attributes’ coefficients. *ε*_*klt*_ ~ *N*(0,*σ*^2^) is an idiosyncratic disturbance term, which is independent of *α*_*k*_ and *γ*_*l*_. According to the central limit theorem, the idiosyncratic error term approaches a normal distribution as the sample size increases. In addition, all error components are independent of ***X***.

Introducing the two-way error components by ***α***_***k***_ and ***γ***_***l***_ is not the only way to specify the panel data model [39]. Some of the other specification alternatives in the error term may include ***η***_***kl***_, which may capture the unobservable effects of origin-destination pairs, or ***η***_***t***_, which captures the unobservable effects of time. Nonetheless, in the context of trip distribution analyses, the influence of origin’s characteristics or destination’s characteristics is of the greatest interest. In addition, the same estimation method proposed in this paper can be adopted to obtain coefficients’ values in those alternative specifications.

Terms ***α***_***k***_ and ***γ***_***l***_ may be estimated in a fixed-effect model, as an alternative to random-effect models. Fixed-effect models can also capture the zonal effects across origin and destination. However, the drawback is obvious: time-invariant explanatory variables would be canceled out in estimation with too many intercepts to be estimated, leading to a loss of degrees of freedom. In addition, fixed-effect models focus on understanding individual zone’s effect, while random-effect settings can empower the model to generalize findings across regions and time.

To further simplify the notation on the spatial autoregressive terms, let ***A*** denote the spatial filterings. Specifically,

$$Ay_{klt}=X\beta +\alpha_{k}+\gamma_{l}+\varepsilon_{klt}$$ (6)

in which

$$\begin{array}{ll} A & =\left(I_{KLT}-\rho_{o}I_{T}⊗\left(W_{o}⊗I_{L}\right)\right)\left(I_{KLT}-\rho_{d}I_{T}⊗\left(I_{K}⊗W_{d}\right)\right)\\ & =I_{KLT}-\rho_{o}I_{T}⊗\left(W_{o}⊗I_{L}\right)-\rho_{d}I_{T}⊗\left(I_{K}⊗W_{d}\right)y_{klt}-\rho_{w}\left(I_{T}⊗W_{w}\right) \end{array}$$ (7)

where ***I***_***KLT***_ is an identity matrix of (*K* × *L* × *T*). The specification implies that *ρ*_*w*_ = −*ρ*_*o*_ × *ρ*_*d*_. While *ρ*_*w*_ can be estimated as an unrestrictive coefficient, the critical interest of this paper is on origin and destination. Hence, a restriction is applied during the model estimation process to ease the computational burden. In addition, the estimation of spatial coefficients requires −1 < *ρ*_*o*_ + *ρ*_*d*_ + *ρ*_*w*_ < 1, which is also set as a restriction throughout this study [26].

Therefore,

$$y_{klt}=A^{-1}X\beta +A^{-1}\left(\alpha_{k}+\gamma_{l}+\varepsilon_{klt}\right)$$ (8)

The expectation of ***y***_***klt***_ is

$$E\left(y_{klt}\right)=A^{-1}X\beta$$ (9)

The variance-covariance matrix of ***y***_***klt***_ is

$$\Omega =A^{-1}\left\{\left(J_{T}⊗I_{K}⊗J_{L}\right)\sigma_{\alpha }^{2}+\left(J_{T}⊗J_{K}⊗I_{L}\right)\sigma_{\beta }^{2}+\left(I_{T}⊗I_{K}⊗I_{L}\right)\sigma_{\varepsilon }^{2}\right\}$$ (10)

where ***J***_*s*_ (*s* = *K*, *L*, *T*) is an (*s* × *s*) dimensional matrix with all elements equal to one.

With the expectation and variance-covariance matrix of ***y***_***klt***_, an attempt of maximum likelihood estimation (MLE) can be executed to obtain the values of coefficients in the random-effect spatial OD dependence model. The likelihood function takes the form:

$$L=\frac{1}{\sqrt{{\left(2\pi \right)}^{KLT}\left|\Omega \right|}}\text{exp}\left\{-\frac{1}{2}{\left(y_{klt}-E\left(y_{klt}\right)\right)}^{T}\Omega^{-1}\left(y_{klt}-E\left(y_{klt}\right)\right)\right\}$$ (11)

### 3.2 Maximum likelihood estimation with spectral decomposition methods

To conduct the MLE, derivations of |**Ω**| and **Ω**^−1^ are required. In panel-data analyses where sizes of matrices are generally large, taking the inverse of large matrices and calculating determinants are time-consuming and sometimes infeasible due to the limited memory of computers. Hence, econometricians often follow the trick devised by Wansbeek and Kapteyn [7] that allows for a simplified calculation of |**Ω**| and **Ω**^−1^. They proposed a simple way to obtain the spectral decomposition (i.e., the eigenvalues and eigenvectors) of the variance components, which are always real symmetric matrices. Then, |**Ω**| and **Ω**^−1^ can be calculated by leveraging the nice features of the spectral decomposition.

Specifically, **Ω**_1_ is the key variable to be derived, which can be defined by

$$\left|\Omega \right|=\left|A^{-1}\right|\left|\left(J_{T}⊗I_{K}⊗J_{L}\right)\sigma_{\alpha }^{2}+\left(J_{T}⊗J_{K}⊗I_{L}\right)\sigma_{\beta }^{2}+\left(I_{T}⊗I_{K}⊗I_{L}\right)\sigma_{\varepsilon }^{2}\right|=\left|A^{-1}\right|\left|\Omega_{1}\right|$$ (12)

$$\Omega^{-1}={\left\{\left(J_{T}⊗I_{K}⊗J_{L}\right)\sigma_{\alpha }^{2}+\left(J_{T}⊗J_{K}⊗I_{L}\right)\sigma_{\beta }^{2}+\left(I_{T}⊗I_{K}⊗I_{L}\right)\sigma_{\varepsilon }^{2}\right\}}^{-1}A=\Omega_{1}^{-1}A$$ (13)

Once |**Ω**_1_| and $\Omega_{1}^{-1}$ are obtained, |**Ω**| and **Ω**^−1^ can be obtained immediately. Initiating the spectral decomposition by a set of transformations, let $\;\bar{J_{i}}=\frac{1}{i}J_{i}$ and $E_{i}=I_{i}-\bar{J_{i}},\;i=K,L,T$. Then,

$$\begin{array}{ll} J_{T}⊗I_{K}⊗J_{L} & =\left(T\bar{J_{T}}\right)⊗\left(E_{K}+\bar{J_{K}}\right)⊗\left(L\bar{J_{L}}\right)\\ & =TL\left(\bar{J_{T}}⊗E_{K}⊗\bar{J_{L}}\right)+TL\left(\bar{J_{T}}⊗\bar{J_{K}}⊗\bar{J_{L}}\right)\\ & =TLN_{010}+TLN_{000} \end{array}$$ (14)

where ***N***_***qrs***_ (*q*, *r*, *s* = 0,1) is a simplified denotation of the Kronecker product of $\bar{J_{i}}$ and ***E***_***i***_.

Similarly,

$$\begin{array}{ll} J_{T}⊗J_{K}⊗I_{L} & =\left(T\bar{J_{T}}\right)⊗\left(K\bar{J_{K}}\right)⊗\left(E_{L}+\bar{J_{L}}\right)\\ & =TK\left(\bar{J_{T}}⊗\bar{J_{K}}⊗E_{L}\right)+TK\left(\bar{J_{T}}⊗\bar{J_{K}}⊗\bar{J_{L}}\right)\\ & =TKN_{001}+TKN_{000} \end{array}$$ (15)

and

$$\begin{array}{l} \begin{array}{l} I_{T}⊗I_{K}⊗I_{L}=\left(E_{T}+\bar{J_{T}}\right)⊗\left(E_{K}+\bar{J_{K}}\right)⊗\left(E_{L}+\bar{J_{L}}\right)\\ =E_{T}⊗E_{K}⊗E_{L}+E_{T}⊗E_{K}⊗\bar{J_{T}}+E_{T}⊗\bar{J_{K}}⊗E_{L}+E_{T}⊗\bar{J_{K}}⊗\bar{J_{L}}\\ +\bar{J_{T}}⊗E_{K}⊗E_{L}+\bar{J_{T}}⊗E_{K}⊗\bar{J_{L}}+\bar{J_{T}}⊗\bar{J_{K}}⊗E_{L}+\bar{J_{T}}⊗\bar{J_{K}}⊗\bar{J_{L}}\\ =N_{111}+N_{110}+N_{101}+N_{100}+N_{011}+N_{010}+N_{001}+N_{000} \end{array} \end{array}$$ (16)

Rearranging **Ω**_1_ in terms of *N* leads to

$$\begin{array}{l} \Omega_{1}=\sigma_{\varepsilon }^{2}\left(N_{111}+N_{110}+N_{101}+N_{100}+N_{011}\right)+\left(TL\sigma_{\alpha }^{2}+\sigma_{\varepsilon }^{2}\right)N_{010}\\ \;+\left(TK\sigma_{\beta }^{2}+\sigma_{\varepsilon }^{2}\right)N_{001}+\left(TL\sigma_{\alpha }^{2}+TK\sigma_{\beta }^{2}+\sigma_{\varepsilon }^{2}\right)N_{000} \end{array}$$ (17)

Define

$$P_{1}=N_{111}+N_{110}+N_{101}+N_{100}+N_{011}$$ (18)

$$P_{2}=N_{010}$$ (19)

$$P_{3}=N_{001}$$ (20)

$$P_{4}=N_{000}$$ (21)

and

$$\lambda_{1}=\sigma_{\varepsilon }^{2}$$ (22)

$$\lambda_{2}=TL\sigma_{\alpha }^{2}+\sigma_{\varepsilon }^{2}$$ (23)

$$\lambda_{3}=TK\sigma_{\beta }^{2}+\sigma_{\varepsilon }^{2}$$ (24)

$$\lambda_{4}=TL\sigma_{\alpha }^{2}+TK\sigma_{\beta }^{2}+\sigma_{\varepsilon }^{2}$$ (25)

As ***P***_***i***_ (*i* = 1…4) are mutually orthogonal and added up to the unit matrix, the variance-covariance matrix Ω_1_ can be written as

$$\Omega_{1}=\lambda_{1}P_{1}+\lambda_{2}P_{2}+\lambda_{3}P_{3}+\lambda_{4}P_{4}$$ (26)

leading to the spectral decomposition. *λ*_*i*_ (*i* = 1…4) are the distinct characteristic roots, i.e., the eigenvalues, of **Ω**_**1**_ with multiplicities (*TKL* − *K* − *L* + 1), (*K* − 1), (*L* − 1), and 1, respectively.

The spectral decomposition has a nice feature that

$$\left|\Omega_{1}\right|=\lambda_{1}^{TKL-K-L+1}\lambda_{2}^{K-1}\lambda_{3}^{L-1}\lambda_{4}$$ (27)

i.e., the determinant of the matrix is the product of all eigenvalues to the exponent of corresponding multiplicities. The other nice feature of the spectral decomposition is that

$$\Omega_{1}^{r}=\lambda_{1}^{r}P_{1}+\lambda_{2}^{r}P_{2}+\lambda_{3}^{r}P_{3}+\lambda_{4}^{r}P_{4}$$ (28)

Hence, $\Omega_{1}^{-1}$ can take the form

$$\Omega_{1}^{-1}=\frac{P_{1}}{\lambda_{1}}+\frac{P_{2}}{\lambda_{2}}+\frac{P_{3}}{\lambda_{3}}+\frac{P_{4}}{\lambda_{4}}$$ (29)

Therefore, the log-likelihood function of the proposed model is given by

$$\begin{array}{ll} LL & =-\frac{KLT}{2}\text{log}\left(2\pi \right)+\frac{1}{2}log\left|A\right|-\frac{1}{2}\text{log}\left|\Omega_{1}\right|\\ & -\frac{1}{2}{\left(y_{klt}-E\left(y_{klt}\right)\right)}^{T}\Omega^{-1}\left(y_{klt}-E\left(y_{klt}\right)\right)\\ & =-\frac{KLT}{2}\text{log}\left(2\pi \right)+\frac{1}{2}\text{log}\left|A\right|-\frac{1}{2}\left(TKL-K-L+1\right)\;\text{log}\;\lambda_{1}-\\ & \frac{1}{2}\left(K-1\right)\;\text{log}\;\lambda_{2}-\frac{1}{2}\left(L-1\right)\;\text{log}\;\lambda_{3}-\frac{1}{2}\;\text{log}\;\lambda_{4}-\\ & \frac{1}{2}{\left(y_{klt}-A^{-1}X\beta \right)}^{T}\left(\frac{P_{1}}{\lambda_{1}}+\frac{P_{2}}{\lambda_{2}}+\frac{P_{3}}{\lambda_{3}}+\frac{P_{4}}{\lambda_{4}}\right)\text{A}\left(y_{klt}-A^{-1}X\beta \right) \end{array}$$ (30)

Given the calculation of the inverse and the determinant of the variance-covariance matrix has been simplified, coefficients of the random-effect spatial OD dependence model can be obtained by maximizing this log-likelihood function using commonly used optimization algorithms.

## 4. Numerical experiments of the proposed estimation approach

A series of simulated time-varying trip distribution datasets are generated for numerical experiments. The number of time points investigated ranges from 3 to 5, and the trip numbers (*Y*) vary at different time points to reflect the time-varying pattern of trip distributions. The proposed MLE is conducted to determine if the true parameter values of the data generating process can be accurately recovered. In addition, these experiments can also examine the relationship between the spatial OD dependences and the unobservable zonal heterogeneity in the proposed model.

The values of explanatory variables can vary over time or be constant. When explanatory variables vary over time, the trip number fluctuations are a force of known interventions and unobservable random effects. When explanatory variables are held constant over time, the implication is that the trip number fluctuation is solely driven by the random effects from the unobservable source. The numerical experiments aim to investigate the former scenario, and the real-world application in Section 5 intends to investigate the latter scenario.

Throughout the numerical experiment, one explanatory variable is defined to capture an intervention with continuous values (e.g., confirmed COVID cases): at least 50 observations in the full simulated explanatory variable are positive integers (under 100), while other observations are defined as zeros. Such a definition means in certain zones across *T* time periods, COVID outbreaks are observed in some traffic analysis zones. The coefficient of this variable can reveal the effect of COVID outbreaks, a passive intervention, on trip distributions. The second explanatory variable is defined as 0–1 to mimic the implementation of a policy (e.g., a certain disease control policy) to prevent the spread of the virus. It could be a wide lockdown or the close of certain public facilities. Its coefficient can show the effect of the active policy intervention on trip distributions.

The spatial weight matrices are initiated by random numbers between 0 and 1 as the values of all elements and then row standardized.

Each simulation scenario contains ten repeating experiments, and each experiment generates a new set of simulated data. To evaluate the performance of the proposed MLE, the average of estimated value (AEV) and the normalized root mean squared error (NRMSE) are proposed:

$$AEV=\frac{\sum \hat{\theta }}{n}$$ (31)

$$NRMSE=\frac{\sqrt{\sum {\left(\hat{\theta }-\theta \right)}^{2}}}{n\times \theta }$$ (32)

where $\hat{\theta }$ is the estimated parameter value, and *θ* is the true parameter value. The denominator *n* is the total number of experiments.

### 4.1 Data size

The proposed MLE method is applied to simulated datasets of different sizes. Table 1 summarizes the estimation results of a series of experiment scenarios with different sizes of simulated datasets. Specifically, scenario 1 assumes 20 origin zones and 20 destination zones observed at four different times. Hence, the dataset size is *K* × *L* × *T* = 20 × 20 × 4 = 1,600. In the spatial autoregressive term, the size of the spatial weight matrix is thus 1,600 × 1,600. A trip distribution study with 20 zones is small enough, if not the minimum meaningful size for a proper analysis: If the proposed MLE can achieve a good estimation for the 20-zone study, it meets the minimum performance requirement in a general sense.

**Table 1:** Numerical experiments of data sizes.

Coef.	True Value	Scenario 1: K, L = 20 T = 4		Scenario 2: K, L = 30 T = 4		Scenario 3: K, L = 20 T = 3		Scenario 4: K, L = 20 T = 5	
		AEV	NRMSE	AEV	NRMSE	AEV	NRMSE	AEV	NRMSE
*ρ*_*o*_	0.3	0.30	0.31%	0.30	0.43%	0.30	0.56%	0.30	0.53%
*ρ*_*d*_	0.3	0.30	0.35%	0.30	0.62%	0.30	0.51%	0.30	0.56%
cons	0.5	0.46	9.56%	0.40	6.93%	0.48	8.99%	0.46	8.87%
*β*_1_	-2	-2.00	0.02%	-2.00	0.05%	-2.00	0.04%	-2.00	0.05%
*β*_2_	1.5	1.50	0.97%	1.49	0.89%	1.51	1.23%	1.47	1.36%
*σ*_*α*_	0.5	0.56	7.20%	0.56	6.20%	0.60	9.32%	0.54	6.62%
*σ*_*γ*_	0.5	0.54	6.72%	0.57	7.97%	0.67	12.02%	0.56	6.53%
*σ*	1	1.00	0.34%	1.00	0.56%	1.00	0.60%	1.00	0.61%

Scenario 2 assumes the size of data is 30 × 30 × 4 = 3,600, which is a notable increase from scenario 1. Based on the experiment results, AEV and NRMSE show only marginal improvements from scenario 1. In other words, the number of origin and destination zones does not play a vital role when the number of zones is greater than 20. When the number of times is 3 (scenario 3) and 5 (scenario 4), the sizes of spatial matrices are 1,200 × 1,200 and 2,000 × 2,000, respectively. The estimation performance, measured in AEV and NRMSE, is similar to scenario 1. The only notable difference is the NRMSE of the two error components, which shows marginal improvements as *T* increases.

Combining the experiment results, the proposed MLE method can successfully recover the parameters in the random-effect spatial OD dependence model. For analysis purposes, scenario 1 is selected as the base scenario to be carried over throughout the rest of the numerical experiments.

The values of *β*_1_ and *β*_2_ may reveal the effects of interventions on trip distribution. If *X*_1_ is the number of confirmed COVID cases varying across time and space, then *β*_1_ means trip numbers would reduce 2 units with 1 additional confirmed case. If *X*_2_ (0–1 variable) indicates whether a park reopens, then *β*_2_ means trip numbers would increase 1.5 units.

### 4.2 Spatial coefficients

Values of spatial coefficients indicate the strength of spatial dependences. Whether the proposed MLE can recover the true values of spatial coefficients is important for analysts as the accuracy of spatial coefficient estimation is important for obtaining the true strength of the spatial autoregressive process and getting an unbiased estimation of explanatory variables’ coefficients.

Furthermore, the proposed random-effect panel-data spatial interaction model specifies two spatial coefficients: One captures the dependence at the origin of trips, and the other captures the dependence at the destination of trips. Meanwhile, the proposed model of this study also specifies two additional error components to characterize individual unobservable effects: One error component characterizes the unobservable effect of the origin, and the other characterizes the unobservable effect of the destination. The interaction of spatial dependences and error components in the model, with respect to origin or destination, is of great interest.

Table 2 summarizes the estimation results of a series of experiment scenarios: each scenario assumes different combinations of spatial coefficients. Scenario 1 assumes the true values of the origin spatial dependence parameter and the destination spatial dependence parameter as 0.3 and 0.3, respectively. Scenario 5–8 assume difference true values as shown in the top row in Table 2.

**Table 2:** Numerical experiments of spatial coefficients.

Coef.	True Value	Scenario 1: *ρ*_*o*_ = 0.3, *ρ*_*d*_ = 0.3		Scenario 5: *ρ*_*o*_ = 0.7, *ρ*_*d*_ = 0.7		Scenario 6: *ρ*_*o*_ = 0.3, *ρ*_*d*_ = 0.7		Scenario 7: *ρ*_*o*_ = -0.3, *ρ*_*d*_ = -0.3		Scenario 8: *ρ*_*o*_ = -0.7, *ρ*_*d*_ = -0.3	
		AEV	NRMSE	AEV	NRMSE	AEV	NRMSE	AEV	NRMSE	AEV	NRMSE
-	-	0.30	0.31%	0.70	0.12%	0.30	0.41%	-0.30	-0.65%	-0.70	-0.34%
-	-	0.30	0.35%	0.70	0.06%	0.70	0.08%	-0.30	-0.93%	-0.30	-0.78%
cons	0.5	0.46	9.56%	0.55	7.83%	0.59	14.84%	0.54	11.03%	0.54	10.45%
*β*_1_	-2	-2.00	0.02%	-2.00	0.03%	-2.00	0.04%	-2.00	0.04%	-2.00	0.05%
*β*_2_	1.5	1.50	0.97%	1.53	1.68%	1.49	1.19%	1.50	1.22%	1.50	1.26%
*σ*_*α*_	0.5	0.56	7.20%	0.87	24.97%	0.89	25.83%	0.44	6.06%	0.42	6.88%
*σ*_*γ*_	0.5	0.54	6.72%	0.95	30.92%	0.57	8.97%	0.42	6.62%	0.33	11.47%
*σ*	1	1.00	0.34%	1.05	1.74%	1.02	1.01%	1.01	0.74%	1.01	0.80%

The estimation of all scenarios has achieved good results, with most NRMSE of parameters within 10% and all MSE are less than 21%. It is worth noting that the NRMSE of *β* is within 1%, indicating the proposed MLE can recover the true values of *β* successfully. Among all scenarios, scenario 1 and scenario 8 have achieved the best results—all NRMSE are less than 11%.

Scenario 5 shows that as the value of spatial coefficients increases, NRMSE of the error components increase. The NRMSE of the origin’s and destination’s error components are 25% and 31%, respectively, when the true values of error components are pre-set as 0.5. This finding reveals that the spatial dependences and the error components, treated as random effects, are strongly associated, as they both capture the unobservable individual effects at the origin or destination of a trip distribution.

Moreover, scenario 6 shows that a greater value of spatial coefficient on one end (origin or destination) of trips is associated with higher NRMSE in the random effects of the other end of trips. In other words, spatial spillovers and the unobservable zonal heterogeneity are associated.

The AEV in scenarios 1, 5, and 6 show that when spatial coefficients are positive, the unobservable zonal heterogeneity tends to be slightly over-estimated. Scenario 7 and 8 show that when spatial coefficients are negative, the unobservable zonal heterogeneity is slightly under-estimated. This finding indicates again that the spatial spillovers and the error components are associated with the estimation.

### 4.3 Error components

The two error components in the specified model characterize the unobservable zonal effects of origin and destination. The values of *σ*_*α*_ and *σ*_*γ*_ are the standard deviations of these unobservable effects. Understanding these standard deviations would help decompose the variation observed in trip distribution data over time and improve the trip distribution models’ fitting. Table 3 summarizes a series of experiment scenarios, and each scenario assumes different values of the standard deviation of error components. Scenario 1 assume the standard deviations of the origin’s unobservable effect and the destination’s unobservable effect as 0.5 and 0.5, respectively. Scenario 9–11 assume different true values as shown in the top row of Table 3.

**Table 3:** Numerical experiments of error components.

Coef.	True Value	Scenario 1:		Scenario 9:		Scenario 10:		Scenario 11:	
		*σ*_*α*_ = 0.5		*σ*_*α*_ = 2		*σ*_*α*_ = 0.5		*σ*_*α*_ = 2	
		*σ*_*γ*_ = 0.5		*σ*_*γ*_ = 2		*σ*_*γ*_ = 2		*σ*_*γ*_ = 0.5	
		AEV	NRMSE	AEV	NRMSE	AEV	NRMSE	AEV	NRMSE
*ρ*_*o*_	0.3	0.32	0.31%	0.31	2.28%	0.32	4.26%	0.32	4.06%
*ρ*_*d*_	0.3	0.32	0.35%	0.30	3.41%	0.32	2.99%	0.32	3.25%
cons	0.5	0.43	9.56%	0.56	47.48%	0.35	24.96%	0.58	28.63%
*β*_1_	-2	-2.01	0.02%	-2.02	0.04%	-2.00	0.05%	-2.00	0.05%
*β*_2_	1.5	1.49	0.97%	1.49	0.45%	1.52	0.56%	1.49	0.43%
*σ*_*α*_	-	0.58	7.20%	2.26	7.14%	0.59	9.09%	2.33	8.73%
*σ*_*γ*_	-	0.56	6.72%	2.34	7.31%	2.23	6.64%	0.56	7.69%
*σ*	1	0.99	0.34%	1.01	0.58%	1.00	0.59%	1.00	0.49%

Scenario 9 pre-sets the standard deviations of error components as 2. The experiment results show that the estimation of standard deviation and spatial coefficients is fairly well in terms of AEV and NRMSE. Nonetheless, NRMSE of the constant term increases dramatically, indicating greater standard deviations of error components disturb the estimation of constant. In fact, the unobservable effects (UE) of origin and destination can be understood by

$$UE_{k}=c_{k}+\alpha_{k}$$ (33)

and

$$UE_{l}=c_{l}+\gamma_{l}$$ (34)

where *c* = *c*_*k*_ + *c*_*l*_. In other words, the unobservable effects of origin and destination consist of a fixed intercept and a random variable to capture zonal heterogeneity. Constant *c* in the final model specification is a combination of the intercepts of unobservable effects of origin and destination. This underlying relationship is further demonstrated in Scenarios 10 and 11. Scenarios 10 and 11 assume that either the origin’s or the destination’s unobservable effect has a standard deviation of 0.5, and the other is 2. The experiment results show NRMSE drops by about half.

## 5. Real-world case study

The proposed random-effect spatial OD dependence model and the MLE with spectral decomposition methods are applied to the empirical trip distribution data collected and processed from cellular signals in Hangzhou, China. Unlike the numerical experiment which attributes time-varying trip distributions to a joint force of known interventions and unobservable effects, this empirical analysis aims to examine a recurring morning-peak traffic distribution solely driven by the random effects from the unobservable source.

### 5.1 Data description

The rapidly increasing ownership of mobile phones in China has enabled transportation analysts to take advantage of cellular signaling data for transportation planning and management. In the capital city of Zhejiang province, Hangzhou, a comprehensive trip distribution dataset has been collected and processed. The data sources include the cellular signals, socio-economic factors, and transportation data of 23 traffic analysis zones along the Qiantang River. These traffic analysis zones cover the most developed regions in Hangzhou, and the main stadium of the 2022 Asian Games is also located in these areas. China Mobile, the largest cellular service provider in China, provided the aggregated level of data of four consecutive morning peak periods. As the data is zonal aggregated, no individual location and demographic information can be derived from the release data.

Table 4 summarizes the statistics of variables in the proposed model. The dependent variable characterizes the time-varying pattern of trip distributions, which is the logarithm of the number of trips between each OD pair during morning peak hours on a specific weekday. In this study, four times are used to constitute the panel, i.e., Tuesday morning, Wednesday morning, Thursday morning, and Friday morning. Hence, with 23 zones, the total number of rows in the model is *K* × *L* × *T* = 23 × 23 × 4 = 2,116. The explanatory variables include population and the important points of interest (POI) to capture trip production and attraction in the trip generation process. Note that a few alternative POI variables have been examined, and the corresponding estimation results have been compared. Finally, the selected POI variable is defined as the squared summation of the number of high-grade commercial centers, hospitals, and transportation hubs. Meanwhile, variables of the length of road lanes, the number of transit lines, and travel time between origin and destination try to capture the transportation accessibility. Setting aside other meaningful variables to explain trip distribution in general or for this particular empirical study, the investigated variables have accounted for the commonly investigated ones in trip distribution analyses.

**Table 4:** Summary statistics of variables in the trip distribution models.

Variable Name	Definition	Mean	Standard Deviation	Max	Min
**Dependent Variables**					
TuesMor	Logarithm of the number of trips divided by 1,000 during Tuesday’s morning peak hours	-2.89	2.28	4.45	-6.91
WedMor	Logarithm of the number of trips divided by 1,000 during Wednesday’s morning peak hours	-2.89	2.27	4.46	-6.91
ThurMor	Logarithm of the number of trips divided by 1,000 during Thursday’s morning peak hours	-3.25	2.83	4.47	-9.21
FriMor	Logarithm of the number of trips divided by 1,000 during Friday’s morning peak hours	-3.20	2.77	4.46	-9.21
**Explanatory Variables**					
Pop_O	Logarithm of population divided by 1,000	1.03	1.26	2.78	-2.14
Pop_D					
POI_O	Squared summation of high-grade commercial centers, hospitals, and transportation hubs	11.17	21.86	100	0
POI_D					
Lane_O	Length of road lanes (km)	0.16	0.47	1.36	-0.87
Lane_D					
Transit	Total number of subway and bus lines	14.99	18.70	110	0
Time	Travel time between origin and destination in minutes	14.95	8.32	37.93	0

The spatial weight matrix is defined by the contiguity of traffic analysis zones. As long as two zones share the same border, the corresponding element in the matrix is defined as 1 and otherwise 0. Then, the matrix is row-standardized to ensure spatial stability and used to evaluate spatial dependences at both the origin and the destination.

### 5.2 Results analysis

The empirical trip distribution data with varying trip numbers over four mornings, are estimated by four different models. Results are shown in Table 5. Model 1 is the full model, accounting for spatial OD dependences and the unobservable zonal heterogeneity simultaneously and the rest are the constrained models. Model 2 assumes no random effects in the data while allowing for spatial OD dependences. Model 3 assumes no spatial dependences while allowing for random effects. Model 4 is the simplest standard linear model without considering spatial OD dependences or random effects.

**Table 5:** Estimation results of trip distribution models.

	Model 1: Full Model		Model 2: Spatial OD Dependences Only		Model 3: Random Effects Only		Model 4: Linear Model	
Variables	Coef.	t-stat	Coef.	t-stat	Coef.	t-stat	Coef.	t-stat
***ρ***_***o***_	0.6632***	27.24	0.9280***	115.67				
***ρ***_***d***_	0.7176***	31.01	0.8842***	66.86				
***σ***_***α***_	0.5231***	6.87			1.0539***	6.90		
***σ***_***γ***_	0.5608***	6.64			1.0098***	6.78		
**Pop_O**	0.0219	0.48	0.0161*	2.03	0.2085+	1.11	0.2710***	6.85
**Pop_D**	0.0637+	1.39	0.0206***	3.32	0.4166*	2.24	0.4791***	12.11
**POI_O**	0.0036+	1.21	0.0006+	1.26	0.0203*	1.81	0.0203***	9.43
**POI_D**	0.0032+	1.02	0.0001	0.45	0.0182*	1.71	0.0182***	8.45
**Lane_O**	0.3401**	2.47	0.1504**	2.76	1.0211*	2.21	1.0986***	11.48
**Lane_D**	0.3535***	3.58	0.1224**	2.62	0.8015+	1.60	0.8790***	9.19
**Transit**	0.0064***	6.77	-0.0002	-0.34	0.0336***	15.49	0.0221***	8.20
**Time**	-0.0152***	-5.20	-0.0105***	-5.41	-0.0297***	-6.42	-0.0558***	-10.24
**constant**	-0.4667***	-6.87	0.0338+	1.33	-4.4811***	-10.37	-4.0725***	-38.79
**Log Likelihood**	-3497.3		-3665.2		-3864.2		-4538.2	

^+^ p<0.15,

* p<0.05,

** p<0.01,

*** p<0.001

Comparing the log-likelihood values which indicate the goodness-of-fit of models, model 1 has the highest value while model 4 has the lowest value. Comparing model 1 against model 2 which has the second-highest log-likelihood value, the improvement is significant. A commonly used statistics, likelihood ratio (LR), can be constructed and tested to justify that the increase is significant:

$$LR=-2\left(LL\left(1\right)-LL\left(2\right)\right)=335.8$$ (35)

With only two additional parameters (the degree of freedom used in the likelihood ratio test), the log-likelihood ratio is 335.8. This is a significant number that can reject the null hypothesis in the chi-square likelihood ratio test, which justifies model 1 presents the best model fitting. In other words, in this empirical trip distribution study, both spatial OD dependences and the unobservable zonal heterogeneity are important and should be captured in a rigorous scientific model.

The random effects are statistically significant in model 1. The finding indicates that the estimation of model 2 is less efficient than model 1 because model 1 better characterizes the variance in trip distribution data. One notable estimated coefficient is the effect of transit. Model 2 finds that transit has no effect on trip distribution, which is neither a reasonable claim in transportation planning theories, nor a valid conclusion given models 1, 3, and 4 show that transit is positively correlated with the trip distribution.

Model 2 and model 3 examine spatial OD dependences and the unobservable zonal heterogeneity individually. Results indicate both effects are significant. Comparing the log-likelihood values, model 2 is significantly higher, indicating spatial OD dependences play a more important role in improving the goodness-of-fit than the unobservable zonal heterogeneity.

In models 1, 2, and 3, the estimated coefficients of *ρ*_*o*_, *ρ*_*d*_, *σ*_*α*_ and *σ*_*γ*_, and the corresponding t-statistics show that all parameters of spatial OD dependences and random effects are statistically significant. Such findings confirm that both spatial OD dependences and random effects are important, and not capturing them would jeopardize models’ performance.

From model 4 to model 1, the t-statistics of estimated coefficients are dropping. The explanatory power of attributes is moved to the unobservable zonal heterogeneity and spatial OD dependences. Unlike the linear model which only uses attributes to explain the variation in the sample trip distribution data, models 1, 2, and 3 assert the variation in trip distribution can be explained by a joint force of attributes, spatial OD dependences, and the unobservable zonal heterogeneity. For example, the variable POI, characterizing the trip attraction, is significant at the 0.001 level in model 4. In model 3, its significance is reduced to the 0.05 level. Hence, instead of being fully confident about controlling POI in the trip distribution management, model 3 reveals that trip distribution is determined by POI with less confidence and actually, the variation of trip numbers is highly dominated by the variation in zonal characteristics. In addition, the spatial OD dependences further drop the significance of POI coefficients to the 0.15 level, and the POI at destination is even insignificant in model 2. Again, the model results imply that entirely relying on adjusting POI for managing trip distribution may lead to erroneous outcomes. Policymakers should consider spatial OD dependences and the unobservable zonal heterogeneity to make more accurate transportation policies.

In summary, the real-world application shows that the proposed model outperforms alternative models. Jointly capturing the spatial OD dependences and unobservable zonal heterogeneity can improve the model’s fitting and offer additional insights into time-varying trip distributions.

It’s worth noting that the interpretation of attributes’ effects can be conducted through variables’ marginal effects. LeSage and Thomas-Agnan [17] explained the calculation methods, and a few studies applied the methods in empirical data analyses [18, 20]. While not being the focus of this paper, marginal effects are not presented here. In addition, the proposed model can reveal the ceteris paribus causal relationship between explanatory variables and trip distributions, which outperforms the data-driven approaches (which are good at prediction) in terms of the explanatory power.

## 6. Conclusions and discussions

This paper proposes a random-effect spatial OD dependence model to investigate time-varying trip distribution data. The proposed model can jointly capture spatial OD dependences and the unobservable zonal heterogeneity and is estimated by a maximum likelihood estimation using spectral decomposition methods. Existing spatial interaction models have not examined spatial OD dependences and the unobservable zonal heterogeneity simultaneously. Given the demand of analyzing time-varying trip distribution in response to the unprecedented pandemic, the research community needs rigorous scientific models to provide additional insights into time-varying trip distribution to lay the groundwork for making more accurate transportation policies.

The contributions of this paper can be summarized into three folds. First, this paper specifies a random-effect spatial OD dependence model, a new model specification to the literature. Second, this paper develops a maximum likelihood estimation with spectral decomposition methods to estimate the proposed model, a new model estimation technique developed upon existing algorithms. Third, this paper applies the proposed model to investigate a set of numerical experiments and an empirical trip distribution analysis that jointly captures spatial effects (spatial interaction and spatial OD dependences) and the unobservable zonal heterogeneity.

While the numerical experiments have shown that the proposed model can effectively evaluate policy interventions, the real-world application does not include explanatory variables varying over time. The proposed specification and estimation methods can be adopted immediately when the real-world data during the pandemic is available, and policy intervention variables are added. Another potential work is to extend the proposed model to capture longer time frames in trip distribution analyses, which may lead to temporal autoregressive processes. Such models can further analyze the response of trip distributions to a societal shock. This would require passively collected OD matrices at more points in time.

## Supporting information

S1 File (XLSX)
